# Editorial Notes

**Published:** 1911-09

**Authors:** 


### Editorial Notes.

**British Medical Association, 1911.**

The British Medical Association had a genial welcome at Birmingham, and the accessibility of the meeting-place led to the presence of a very large gathering. Much interest was felt in the remarkable work of the municipality and University and the arrangements of the many local institutions. The city, under the Extension Act, will shortly have nearly 900,000 inhabitants, and has long taken a lead in municipal enterprise. A very interesting history of the Medical School was given in the handbook, starting with the first lectures of Sands Cox in 1825 down to the present arrangements for the control of clinical teaching in the hospitals and the affiliation of the special institutions, which shows the great difficulties which have been successfully overcome. The work and position of the local dispensary was the subject of a paper in the Sociological section. Well-to-do working-men have subscribed largely, and therefore claim the right to the use of the funds which were said to have been intended for the very poor. Indeed, the difficulty of restricting out-patient departments to their proper purposes is as great here as elsewhere. The probable effect of the Insurance Bill on this institution and hospitals generally led to an extraordinary variety of papers. Mr. Neville Chamberlain thought that just as a grant is made to satisfactory Universities, so a grant should be made to voluntary hospitals which do good work. In the address on surgery the conversion of the hospitals into State-managed institutions was pointed to as desirable, while Dr. Lauriston Shaw showed the hopeful future of voluntary institutions, especially if the present out-patient departments are restricted to consultations and special treatment.

Few papers of great general interest seem to have been brought forward. The attack on the undue expensiveness of modern surgical methods will probably lead to much discussion, and the debate on pyrexia without a recognised cause showed that the subject was one of importance whether it points to some source of hæmolysis or whatever the explanation be.

The various rooms for the meetings of the sections and for the Pathological Museum were under one roof, close to the hall where the general addresses were given and the representative body met. It goes without saying that this was an immense advantage, especially in the tropical weather we experienced.

The exhibition was a little farther off, in Bingley Hall, but the care of the organisers had provided a free service of motors, which were in constant use. Indeed, few cities are so conveniently situated for a meeting and have such spacious halls and meeting rooms close to the hotels and stations. The magnificent Municipal Buildings, where the Lord Mayor gave us a hospitable reception, was within a stone's-throw, and the Coronation illuminations were relighted for the occasion. The only distant function was that at the new University buildings, some two miles away.

The local committee, who were unwearied in their exertions, provided at the Theatre Royal a charming representation of one of Rostand's plays in English verse, and a most enjoyable smoking concert in the Drill Hall. The blazing sun made the numerous garden parties a great attraction, and in the beautiful recreation grounds of Bournville an outdoor entertainment was enjoyed by about one thousand visitors in ideally perfect weather.

The visits to the factories for which Birmingham is famous were not the least interesting things of the week. The B.S.A. works for rifles and cycles, the pen and pin factories, and many others attracted crowds of visitors, while Stratford, Hereford, Droitwich and Malvern ended a memorable week.

**Small-pox amongst the Basutos.**

*The Transvaal Medical Journal* (June 11th, 1911) contains an article by one of our old Bristol students (Dr. H. A. Spencer) on "The Practice of Inoculation of Small-pox in Basutoland." He found that a man, about 70 years of age, whom he had vaccinated on three different annual visits failed to give any positive result. On inquiry it was found that the old man had a very distinct mark of inoculation just above the patella, and he stated that an operation had been performed upon him when a lad just big enough to join his tribe in battle. In many other unsuccessful cases of vaccination it was ascertained that the failure of the vaccination was due to the fact that inoculation in early life had been the practice whenever small-pox was known to be in the country. On further inquiry it was learnt that inoculation was introduced by the Portuguese of the East Coast at the time when it was in general practice throughout Europe. How the inoculation was accomplished forms an interesting story by Dr. Alexander Spencer in the *Journal* above mentioned.

**John Beddoe, F.R.S.**

We wish to call attention to our obituary notice (page 284), and also to one of our book reviews (page 257), probably the last which the venerable physician and anthropologist, Dr. Beddoe, wrote. His account of the Jews will be of especial interest as giving the mature judgment of one whose career had given an especial opportunity of forming conclusions on controversial points of this kind.

**Royal Commission on Tuberculosis.**

The announcement of Professor Koch at the International Congress in London in 1901 that the tuberculous disease of cattle was not the same thing as tuberculosis of the human subject, and that tubercle bacillus found in the tuberculosis of cattle was non-virulent for man, has undoubtedly greatly delayed legislation on the food supply, and has accordingly tended to diminish the rapidity of the decline of mortality from tuberculosis. The conclusions now announced by the Commissioners in their final report will no longer give any excuse for the postponement of measures to ensure a supply of pure milk, and it is understood that steps are being taken to bring about this result without delay. In this way may be averted the constantly-recurring catastrophe of the transmission of latent bovine tuberculosis to children in early life, which so frequently becomes the origin of cases of pulmonary phthisis in later years.

**Medical Officer of Health.**

We wish to congratulate the staff of the Health Department of Bristol on the happy thought which led them to express their appreciation of the valuable services of Dr. D. S. Davies on the completion of his twenty-five years as Medical Officer of Health. Dr. Davies' professional colleagues are delighted to see that such valuable service as he renders the community is fully recognised and acknowledged. We ourselves are often indebted to him for assistance in the *Journal* work.

